# Review of Teratogenic Effects of Leflunomide, Accutane, Thalidomide, Warfarin, Tetracycline, and Angiotensin-Converting Enzyme Inhibitors

**DOI:** 10.7759/cureus.50465

**Published:** 2023-12-13

**Authors:** Raegan B Abadie, Camryn L Keller, Nicholas T Jones, Erin L Mayeux, Rachel J Klapper, Lillian Anderson, Adam M Kaye, Shahab Ahmadzadeh, Giustino Varrassi, Sahar Shekoohi, Alan D Kaye

**Affiliations:** 1 School of Medicine, Louisiana State University Health Sciences Center, Shreveport, USA; 2 Department of Radiology, Louisiana State University Health Sciences Center, Shreveport, USA; 3 Department of Anesthesiology, Louisiana State University Health Sciences Center, Shreveport, USA; 4 Department of Pharmacy Practice, Thomas J. Long School of Pharmacy and Health Sciences University of the Pacific, Stockton, USA; 5 Pain Medicine, Paolo Procacci Foundation, Rome, ITA

**Keywords:** category x, deformities, acei, tetracycline, warfarin, thalidomide, accutane, leflunomide, teratogenicity

## Abstract

Teratogenic agents have been shown to have drastic and detrimental effects on fetuses if exposed to the agent during uterine life. The most sensitive time for a developing fetus is during the first trimester, and teratogenic exposure during this time can lead to severe deformities in the fetus. The Food and Drug Administration has categorized teratogenic agents based on the severity of their effect on the fetus; these categories include A, B, C, D, and X. Category A is the safest, with the most dangerous, and highly contraindicated in pregnant patients being Category X. This review article will discuss the teratogenic agents leflunomide, isotretinoin, thalidomide, warfarin, tetracycline, and angiotensinogen-converting enzyme inhibitors. Leflunomide can cause cranioschisis, exencephaly, and vertebral, head, and limb malformations. Isotretinoin's main teratogenic effects include central nervous system malformations, hydrocephalus, eye abnormalities, cardiac septal defects, thymus abnormalities, spontaneous abortions, and external ear abnormalities. Thalidomide has been shown to cause limb deformities, bowel atresia, and heart defects when the embryo is exposed to the agent during development. Warfarin can lead to spontaneous abortion and intrauterine death, as well as nasal hypoplasia, hypoplasia of extremities, cardiac defects, scoliosis, and mental retardation when exposed in utero. Tetracycline’s teratogenic effects include gastrointestinal distress, esophageal ulceration and strictures, teeth discoloration, hepatotoxicity, and calcifications. Angiotensinogen-converting enzyme inhibitors can cause skull hyperplasia, anuria, hypotension, renal failure, lung hypoplasia, skeletal deformation, oligohydramnios, and fetal death. Teratogenic effects can be avoided if the pregnant patient is educated on the teratogenic effects of these agents.

## Introduction and background

A teratogen is an agent that can affect the normal development of an embryo or fetus during the developmental period. Teratogenicity of drugs can produce a range of effects depending on which developmental stage the fetus was in when exposed to the teratogen, how long the fetus was exposed, and the dosage of exposure. The most sensitive and likely developmental period to develop deformities due to teratogens is around 14 to 60 days post-conception. This is because it would be after the fertilized egg has implanted and when the most important processes are beginning to take place in the fetus's development including the division and multiplication of cells to create the beginning components for the future development of a fetus [1]. 

The Food and Drug Administration (FDA) has prioritized identifying teratogenic agents and has developed a system for defining the teratogenic risk of drugs by analyzing data from human and animal studies. These categories include A, B, C, D, and X. The safest category of a teratogenic agent is category A, and the most dangerous and contraindicated for use in pregnancy is category X. Category A is described as “Adequate, well-controlled studies in pregnant women have not shown an increased risk of fetal abnormalities.” Category B is described as “Animal studies have revealed no evidence of harm to the fetus; however, there are no adequate and well-controlled studies in pregnant women. Animal studies have shown an adverse effect, but adequate and well-controlled studies in pregnant women have failed to demonstrate a risk to the fetus”. Category C is listed as “Animal studies have shown an adverse effect and there are no adequate and well-controlled studies in pregnant women. Or No animal studies have been conducted, and there are no adequate and well-controlled studies in pregnant women”. Category D is “Studies, adequate well-controlled or observational, in pregnant women have demonstrated a risk to the fetus. However, the benefits of therapy may outweigh the potential risk”. Category X is "Studies, adequate, well-controlled, or observational, in animals or pregnant women have demonstrated positive evidence of fetal abnormalities. The use of the product is contraindicated in women who are or may become pregnant."

The present investigation includes class C teratogenic drugs, including angiotensinogen-converting enzyme (ACE) inhibitors when used in the first trimester, class D teratogenic agents, ACE inhibitors in second and third trimesters, warfarin, and tetracycline, as well as class X teratogenic agents that cannot be used in pregnancy, including leflunomide, Accutane, and thalidomide [2-9].

## Review

Arava (leflunomide) overview

Leflunomide (Arava) is an isoxazole derivative that targets lymphocytes and prevents their proliferation, which reduces immune system activity [3,6]. Leflunomide must be converted to its active metabolite once ingested, and the active metabolite is a noncompetitive inhibitor of the enzyme dihydro-orotate dehydrogenase, which is necessary for DNA synthesis. By inhibiting the production of DNA, the drug halts the proliferation of lymphocytes such as B and T cells [4]. Reducing the number of circulating immune cells is useful in the treatment of autoimmune conditions, which are diseases caused by an overactive immune system producing antibodies to tissues within the body. This includes systemic lupus erythematosus, rheumatoid arthritis, and myasthenia gravis [3]. 

Leflunomide can cross the placenta and cause teratogenic effects, which have been seen in both rabbit and rat models. These teratogenic effects include cranioschisis and exencephaly when given during days seven to 17 of pregnancy. Additionally, multiple head, vertebral, and limb malformations can occur along with incomplete ossification of bones. One study that tested the teratogenic effects of leflunomide on rats concluded that the 70 mg/kg dose is the most toxic and leads to all fetuses being lost. In addition, when given at a 30 mg/kg dose, the number of malformations due to intrauterine growth restriction increased significantly, but fewer fetuses were lost [5]. To prevent these teratogenic effects, women may be advised to switch to a different disease-modifying antirheumatic drug (DMARD) with a less harmful teratogenic profile. Because the active metabolite of leflunomide has an extremely long half-life, women are advised to stop taking the drug prior to conception to ensure adequate elimination and reduce the risk of teratogenic effects. However, some women experience fewer rheumatoid arthritis symptoms during pregnancy due to changes in the immune system, so these patients may be able to stop treatment with leflunomide completely [4].

Accutane overview

Isotretinoin (Accutane) is a retinoid compound that affects cellular growth, differentiation, immunomodulation, and malignant potential of cells by affecting DNA transcription. Retinoids bind to nuclear retinoid receptors, which are related to the DNA transcription factors, and retinoid receptors can also affect DNA transcription by binding to DNA regulatory regions called hormone-response elements. Once bound, isotretinoin can act similarly to steroid hormones, vitamin D, and thyroid hormones. Isotretinoin is used to treat cutaneous acneiform eruptions by causing a reduction in the number and size of sebaceous glands, reducing the activity of androgens on these glands. Additionally, isotretinoin affects keratinocyte maturation and adhesion, preventing comedone formation, and the drug reduces the inflammatory response involved in acne formation. Isotretinoin is also useful in the treatment of rosacea, folliculitis, and persistent facial edema [6].

Retinoids, including isotretinoin, are extremely potent teratogens that cause a distinct pattern of symptoms called retinoic acid embryopathy. This constellation of symptoms includes central nervous system malformations such as hydrocephalus, external ear abnormalities, eye abnormalities, cardiac septal defects, and thymus gland abnormalities, and it can also increase the risk for spontaneous abortions. In addition, studies have shown that up to 60% of children with isotretinoin exposure have neurocognitive impairment even if they may not show physical symptoms. These effects have been found in approximately 25% of babies with isotretinoin exposure, so patients are encouraged to completely stop treatment before becoming pregnant and cannot receive alternative treatment with other retinoids [7].

Because of the high teratogenicity rate, multiple precautionary measures are taken before prescribing isotretinoin. These precautions include physicians ensuring that the patient is aware of harmful adverse effects and patients using two different forms of contraception before and during treatment. Once isotretinoin therapy has been completed, it is only detectable in the body for ten days, so there is no longer a risk of teratogenic effects [7,8]. 

Thalidomide overview

Thalidomide (THD) is one of the most teratogenic medications responsible for an epidemic that is attributed to over 10,000 children born worldwide with severe birth defects in the short time it was used to treat the morning sickness of pregnant patients [9]. THD has several pathways that it affects, all contributing to the teratogenic effects. THD exerts its effects by binding to celebron and inhibiting the CRL4CRBN E3 ubiquitin ligase activity, which typically targets intracellular molecules for destruction. This specific mechanism of action leads to the teratogenic effects of limb deformities in THD in the fetus, as it is proposed that this interferes with the normal molecular signaling that occurs during embryogenesis [9,10]. Most THD survivors have a condition that results in missing limbs that result in the hands or feet being attached to the trunk of the body and additional malformation of the digits. Due to the effects of THD on tissues of various body systems and other THD pathways, a large proportion of babies have bowel atresia and heart defects, resulting in their death within the first year of life [9]. Since its cessation of use in pregnant patients, has become a category X teratogenic and an FDA black box warning; therefore, this drug should not be used in pregnant patients, and contraceptives should be initiated if a patient is prescribed this medication for other conditions.

Related to the selective inhibition of tumor necrosis factor-α (TNF-α), a proinflammatory mediator, thalidomide can be used to treat several inflammatory conditions such as AIDS-related aphthous stomatitis, erythema nodosum leprosum, graft-versus-host disease, multiple myeloma, systemic light chain amyloidosis, and Waldenström's macroglobulinemia [11,12]. Recent literature refers to the actions of THD against certain cancers due to its anti-angiogenic potential, which is a celebron independent mechanism [9,12]. Although THD was responsible for a preventable epidemic, a stain in medical history, it can have a future as a treatment for various sold-tumor cancers, autoimmune diseases, and inflammatory diseases.

Two thalidomide analogs, pomalidomide and lenalidomide, were agents developed to have decreased side effects known to cause teratogenic effects. Both exert their anti-myeloma effect by downregulating tumor cells, affecting cell adhesion, and reducing pro-survival cytokines. Lenalidomide is used for the treatment of relapsed plasma cell myeloma and myelodysplastic syndromes associated with the deletion of 5q [13]. Lenalidomide has a distinct adverse effect profile when compared to thalidomide, including myelosuppression. Pomalidomide is given to patients who have failed lenalidomide and bortezomib therapy and has been shown to have benefits in these patients [14]. Pomalidomide can also be used in relapsed plasma cell myeloma and myelofibrosis [13]. However, since both lenalidomide and pomalidomide are analogous to thalidomide, they can result in teratogenic effects and should, therefore, be avoided in pregnancy.

Warfarin overview

Clinically, warfarin is used as an anticoagulant, with the most common causes for warfarin use being heart valve replacement and deep venous thrombosis history in patients [15]. Several coagulation facts undergo post-translation modifications in the liver, requiring the vitamin-K-dependent gamma-carboxylation of inactive precursors to function correctly in the serum. During this process, the vitamin-K cofactor becomes oxidized into vitamin-K epoxide, which is inactive. Vitamin-K epoxide reductase reduces this oxidized cofactor back into its active form, but it is also the target for warfarin. Therefore, warfarin inhibits the production of vitamin-K-dependent coagulation factors, causing a clinical increase in prothrombin time (PT), partial thromboplastin time (PTT), and the international normalized ratio (INR). This mechanism of action explains why vitamin K supplementation can also decrease and reverse the effects of warfarin [16].

Warfarin is classified as a category X drug by the FDA, meaning it is contraindicated in pregnancy because it can cross the placenta and cause birth defects in the fetus. Additionally, maternal warfarin use can lead to spontaneous abortion and fetal death [17]. The use of warfarin during pregnancy causes a constellation of symptoms called fetal warfarin syndrome, the most prominent being nasal hypoplasia, hypoplasia of the extremities, cardiac defects, scoliosis, and mental retardation. If a patient is pregnant or plans to become pregnant, the recommended anticoagulant is heparin or low-molecular-weight heparin (LMWK), as neither crosses the blood-brain barrier [5,7]. The highest risk of teratogenicity for warfarin use during pregnancy is usage between six to nine weeks of fetal gestation, but the dosage of the medication with teratogenicity rates is unstable. Birth defects can be caused with as low as 5 mg/day of warfarin during the six-to-nine-week window of pregnancy [17]. Since warfarin is a teratogenic drug, it is advised to counsel patients of the risks of becoming pregnant while on the medication and offer pregnancy test for eligible patients at the initiation of warfarin use. According to a 2020 study, there is inconsistent management across healthcare risk management and advising patients for warfarin use [18].

Tetracycline overview

Tetracyclines, commonly used to treat spirochete infections, actinomycosis, chlamydial infections, pelvic inflammatory disease, syphilis, acne, and Whipple disease, to name a few, are a class of broad-spectrum antibiotics. They include naturally occurring (tetracycline, chlortetracycline, oxytetracycline, demeclocycline) and semi-synthetic (lymecycline, metacycline, minocycline, and doxycycline) varieties. Their mechanism of action is described as “bacteriostatic” as they halt proper functioning and replication. This is done by inhibiting the 30S ribosomal unit such that aminoacyl-tRNA cannot bind to the acceptor site on the mRNA-ribosome complex. Certain bacterial strains have developed resistance to tetracyclines, reducing efficacy [19]. Based on pharmacokinetic considerations, doxycycline is the preferred agent [20].

The dosing of tetracyclines is different for children and adults. Adults can receive 1 g/day or up to 2 g/day for severe infections. Pediatric patients can receive 25 mg/kg/day to 50 mg/kg/day when split into four equal doses. Therapeutic concentrations are maintained through dosing two to four times a day. This can be impaired with antacids or dairy consumption due to chelation and disrupted gastrointestinal (GI) absorption.

Common adverse effects include GI distress, esophageal ulceration and strictures, and teeth discoloration in children under eight. Rare instances of hepatotoxicity and exacerbation of renal failure have been reported [1]. A few teratogenic effects have been reported, leading to contraindications. Drug ingestion after the fourth month of pregnancy can lead to grey/yellow discoloration of “baby” teeth without alterations in development or enamel, calcification, and reduced growth of bones and teeth. Normal bone growth is resumed after the offending agent is stopped [21].

New evidence shows that tetracycline usage during pregnancy has correlative relationship changes in maternal thyroid hormones, including free thyroxine (FT4), thyroid stimulating hormone (TSH), total triiodothyronine (TT3), and total thyroxine (TT4), during the first trimester, specifically [22]. This includes a positive association with TSH and TT3 and a negative association with FT4 and TT4. In conclusion, the lack of research and reported data limits understanding of tetracycline’s overall contribution to maternal thyroid hormone changes.

According to the FDA, tetracyclines have been placed into the pregnancy risk category D. This indicates there is evidence (from human studies) supporting the contraindication of the drug during pregnancy due to its adverse fetal effects, yet the benefits may warrant the use in a pregnant woman if deemed necessary [23]. Therefore, the evidence indicating tooth discoloration and reduced bone growth demonstrates the teratogenic effects of tetracycline use, limiting its indication during pregnancy.

Angiotensin-converting enzyme inhibitors overview

ACE inhibitors are used in the management and treatment of hypertension. The use of these drugs helps decrease the associated risks of hypertension, including coronary disease, heart failure, stroke, diabetes, and other cardiovascular conditions. ACE inhibitors lower the mean arterial blood pressure and systolic and diastolic blood pressure, not limited to just hypertensive patients. This is due to their ability to blood angiotensin-converting enzyme and the subsequent conversion of angiotensin I to angiotensin II. Other effects include decreasing afterload, preload, systolic wall stress, and increased cardiac output. Reduction of aldosterone and antidiuretic hormone production allows for increased salt excretion. According to the American Heart Association/American College of Cardiology (AHA/ACC), ACE inhibitors are recommended as the first-line anti-hypertensive treatment.

Except enalapril, which is given intravenously, all ACE inhibitors are given orally. Titration cannot exceed 5 mg IV every six hours for enalapril and is recommended to be 0.625 to 1.25 mg every six hours. Other dosing is dependent on which drug is administered. Dosage should be decreased in those with heart failure, salt depletion, or renal impairment. Monitoring must be done to check for increased potassium, glomerular filtration rate (GFR) drop, and creatinine.

Adverse effects include a dry, nonproductive cough and angioedema of the intestines, tongue, glottis, and larynx. This can lead to airway obstruction and compromise. Reversible decline in kidney function has also been reported in people with heart failure and those with pre-existing renal insufficiency. Hyperkalemia, cholestatic jaundice, and hepatitis have also been noted.

ACE inhibitors are contraindicated in pregnancy, having caused skull hypoplasia, anuria, hypotension, renal failure, lung hypoplasia, skeletal deformation, oligohydramnios, and death [24]. A 1998 article in the Lancet has suggested that due to the severe long-term effects, with the inclusion of fetal death, ACE inhibitors should be contraindicated in women of childbearing age. Very early exposure has not shown harmful effects, but caution has still been suggested due to a lack of pregnancy awareness in the early weeks. Fetotoxic effects are observed in the second and third trimesters [25]. 

According to the FDA, ACE inhibitors have been placed into the pregnancy risk category D [24]. According to a 2021 study in the American Society of Health-System Pharmacists, prescribed ACE inhibitor and angiotensin receptor blocker (ARB) rates remained high even with known teratogenic effects. Of the 3,045 patients (females aged 18-49) included in the study population, 37.6% were prescribed ACE inhibitors or ARBs, with only 48% of those women having documented contraception [26]. Overall, the teratogenic effects of ACE inhibitors have been well studied and documented for decades, yet pregnancy prevention for female patients on these anti-hypertensive medications has been limited. 

Of note, ARBs effects are similar to ACE inhibitors in that they act on the RAAS system, however, ARBs antagonize receptor binding of angiotensin II to angiotensin I receptors. ARBs, especially losartan, can cause teratogenic effects. ARBs can cause incomplete ossification of skull bones, transient oliguria, and feed intolerance in a newborn if they had in-utero exposure to losartan; therefore, they should also be avoided in pregnancy [27]. Even though ARBs can be useful in the treatment of hypertension, one who is treating a pregnant mother should prescribe another class of anti-hypertensive drugs to decrease teratogenic risks in the fetus [28,29].

Clinical trials of teratogenic medications and outcomes

There are lots of clinical trials that are currently being done and that have been done to determine the teratogenicity of different medications. This is because teratogenicity and its effects on a fetus are avoidable. So, it is urged to learn more about teratogenic medications to minimize teratogenic effects on fetuses. Below in Table 1 are previous clinical trials assessing the teratogenicity of the drugs described in this review.

**Table 1:** Clinical trials of teratogenic medications and outcomes. CNS: central nervous system

Study	Drug	Groups Studied and Intervention	Results and Findings	Conclusions
DiPaolo 1963 [30]	Thalidomide	Sixty-two female pregnant mice were given various doses of thalidomide, and the defects and litter number were recorded.	Fetal malformations were found at statistically significantly higher levels, and the treated groups had lower litter numbers when compared to the controls. The highest teratogenicity occurred at 62 mg/kg, with treatment beginning in the seventh week and ending in the 11th week, with five out of five fetuses being born with severe birth defects.	With the high incidence of severe birth defects in mine, thalidomide should not be used in pregnant patients.
Fukushima et al. 2007 [5]	Leflunomide	Mice were given various doses of leflunomide, and the number of normal fetuses, dead fetuses, and fetuses with malformations was counted. Additionally, the type of malformations was also recorded.	Fetal malformations were found with all doses of leflunomide, but severe teratogenicity began at the 30 mg/kg dose. The major malformation was exencephaly due to abnormal neural tube development. All fetuses died at the 70 mg/kg dose.	Leflunomide has been shown to have severe teratogenic effects in mice, so it should not be used in expecting mothers because this risk may carry into the human species.
Zomerdijk et al. 2014 [31]	Accutane (isotretinoin)	203,962 pregnant females who were either exposed to isotretinoin 30 days either before or during pregnancy.	9.4% of pregnancies in women exposed to isotretinoin resulted in adverse effects on the fetus.	Isotretinoin carries an increased risk of congenital malformation when mothers are exposed before or during pregnancy.
Basu et al. 2016 [32]	Warfarin	Over a four-year period, 15 mothers delivered babies while on low-dose warfarin therapy (<5 mg/day).	One of the 15 pregnancies resulted in a miscarriage, and four of the babies showed symptomology of fetal warfarin syndrome.	In conclusion, warfarin at any dosage should be avoided in pregnant women.
Bateman et al. 2017 [33]	Angiotensin-converting enzyme inhibitors (ACEI)	Cohort study analyzed 18,515 pregnancies, 2,631 of which were exposed to angiotensin-converting enzyme inhibitors during the first trimester, with the other 15,884 having untreated hypertension.	Overall malformations in the angiotensin-converting enzyme inhibitor exposed group were 5.9% versus 3.3% in the unexposed. Adjusted analysis for confounding factors, such as diabetes, did not suggest an increase in the risk of malformations associated with the first trimester.	Although the evidence does not suggest an increase in malformations due to angiotensin-converting enzyme inhibitors exposure (overall, cardiac, or CNS), it is still important to discontinue Angiotensin-converting enzyme inhibitors prior to the start of the second trimester to prevent fetopathy.
Hill et al. 2021 [34]	Tetracycline	Using the tetracycline on/off system and administering 0.5 mg/ml doxycycline in the drinking water, mice were monitored from embryonic day 15.5 to day 28 post-natal and from day 21 to 63 postnatal.	Developmental exposure to doxycycline led to lasting effects on the gut morphology and weight of the offspring.	Doxycycline shows teratogenic effects regardless of genetic manipulation due to shifts in homeostatic baseline and metabolism. Should be avoided during pregnancy

## Conclusions

The most sensitive time for a fetus in development is during the first trimester, especially 14 to 60 days after conception, as teratogenic exposure during this time can lead to extreme deformities in the fetus, especially with drugs including leflunomide, Accutane, thalidomide, warfarin, tetracycline, and ACE inhibitors. Because of the effects of these medications, extreme caution must be used when considering the use of these in pregnant mothers. Teratogenic agents have a multitude of effects on the fetus’s development depending on when the fetus was exposed, for how long, and the dosage at exposure.

There are currently five categories of teratogenic agents, as defined by the FDA; these categories include A, B, C, D, and X. Category A has the least teratogenic effect, while Category X has the most teratogenic effect, and should never be used in pregnant patients. Leflunomide, isotretinoin, thalidomide, warfarin, tetracycline, and ACE inhibitors are all examples of teratogenic agents in categories C, D, and X. As discussed previously, leflunomide can cause cranioschisis, exencephaly, and vertebral, head, and limb malformations. Isotretinoin's main teratogenic effects include central nervous system malformations, hydrocephalus, eye abnormalities, cardiac septal defects, thymus abnormalities, spontaneous abortions, and external ear abnormalities. Thalidomide has been shown to cause limb deformities, bowel atresia, and heart defects when the embryo is exposed to the agent during development. Warfarin can lead to spontaneous abortion and death of the fetus, as well as nasal hypoplasia, hypoplasia of extremities, cardiac defects, scoliosis, and mental retardation when exposed in utero. Tetracycline’s teratogenic effects include gastrointestinal distress, esophageal ulceration and strictures, teeth discoloration, hepatotoxicity, and calcifications. ACE inhibitors have the teratogenic effects of skull hyperplasia, anuria, hypotension, renal failure, lung hypoplasia, skeletal deformation, oligohydramnios, and death in the fetus. With the proper education on teratogenic agents and their effects, a mother can prevent her fetus from developing these severe developmental abnormalities if she avoids these agents, especially during the high susceptibility period. With the recent categorization of drugs and the importance of education about the harmful effects of these medications in pregnancy, it is aiming towards having a future with less teratogenic effects due to these specific drugs. 
